# Exploring the health challenges of affected people in the 2023 Khoy earthquake: a content analysis

**DOI:** 10.1186/s12873-024-01114-7

**Published:** 2024-10-28

**Authors:** Masumeh Akbarbegloo, Ahad Heydari, Mahnaz Sanaeefar, Saeed Fallah-Aliabadi

**Affiliations:** 1 Pediatric Nursing Department, Faculty of Nursing, Khoy University of Medical Sciences, Khoy, Iran; 2 Department of Health in Disaster and Emergencies, School of Medicine, Kurdistan University of Medical Sciences, Sanandaj, Iran; 3 Department of Health in Emergencies and Disasters, School of Public Health, Shahid Sadoughi University of Medical Sciences, Yazd, Iran; 4 Trauma Research Center, Shahid Sadoughi University of Medical Sciences, Yazd, Iran

**Keywords:** Earthquake, Health challenges, Content analysis

## Abstract

**Background:**

An earthquake has significant effects on health and livelihood of people. It is important to identify health needs and challenges of earthquake victims and use them to prepare for other possible earthquakes. Therefore, the purpose of this study is to explain the challenges and health needs of earthquake victims in Iran.

**Methods:**

This was a qualitative study with 25 participation who were affected by the earthquake in Khoy City, and were selected using purposive sampling by snowball method technique in 2023. The study data was collected through open and semi-structured interviews. To analyze the data, the conventional content analysis with an inductive approach was used.

**Results:**

The results show two main categories. The main categories “Management as a missing link in unexpected events” includes 9 subcategories: “The challenge of access to emergency resources and health facilities”, “Feeling of abandonment in medical personnel”, “Weakness in the structural safety and infrastructure of the health system”, “Logistical challenges”, “Risk management training”, “Crisis response management challenges”, “Weakness in the intelligent relief system”, “Management of secondary incidents”, and “Need to provide medical services and disease prevention”. Also, the main categories “Emotional actions of people in crisis” consist of 5 subcategories: “Overexcitement”, “Psychological vulnerability of children”, “Physical complaints caused by stress”, “Confusion caused by the lack of reliable information sources” and “Negative effects of living together with several families”.

**Conclusion:**

To help deal with threats and other challenges in the earthquake crisis, countries should try to improve their capacity to manage natural disasters.

**Supplementary Information:**

The online version contains supplementary material available at 10.1186/s12873-024-01114-7.

## Background

 Earthquakes are one of the most influential geological processes and are one of the most unpredictable natural disasters that have the potential to cause destructive effects on humans and structures [1]. Every year, on average, 3 million people become homeless after the occurrence of natural disasters, and about 80% are related to people whose houses were destroyed due to earthquakes [2].

Due to its location on the Alpine-Himalaya earthquake belt, Iran is one of the 10 major landlocked countries in the world, where 13 major earthquakes have occurred in the past three decades [3]. Also, Iran is the fourth country in Asia in terms of landlessness. Mountainous areas and the Zagros region of Iran are at greater risk of earthquakes [4]. Statistics show that on average, every four years, a severe earthquake occurs in Iran, the result of which is the destruction of 97% of residential units in the area where the earthquake occurred, therefore, it is essential to prepare for this natural phenomenon [5].

Disasters anywhere in the world have significant effects on the health and safety of society, people’s living conditions, and protective infrastructures [6]. Due to the destruction of existing facilities and installation in the region, a very high number of casualties in a short period, need to CPR and lack of quick access to healthcare facilities, the earthquake can have various consequences, including high mortality, trauma to body parts, imposing high costs over time [7], disorders, social and psychological threats to the health that lead to the aggravation of diseases and injuries [8, 9].

On the other hand, in unexpected incidents, many problems and issues occur, such as limited facilities, lack of relief forces, and trained staff in EMS and hospitals, which require the services and power of volunteers [10].

According to the above, it is clear that the earthquake has the potential to create a chain of events that significantly affects public health and can also cause adverse conditions such as death, physical and mental disability, change the course and life path of millions of people and cause severe financial damage to individuals and governments in the affected communities [11].

Emergencies following disasters often occur in developing countries that do not have adequate public health infrastructure, basic medicines and equipment, and adequate numbers of health workers. Vulnerability in developed countries may also increase due to urban environments dense with population and refugees and immigrants with poor economic and social status [12].

Health aid is one of the most important and first aid that reaches disaster areas. Although until recent years, health policymakers focused more on physical complications in the face of traumatic events, but nowadays, the importance of quick and timely treatment of the psychological and social reactions of people affected during an earthquake has become clear to healthcare providers. For this reason, dealing with the health challenges of survivors to normalize reactions and prevent the occurrence of long-term complications that lead to a decrease in the quality of life and efficiency of people, is considered one of the most important goals of the activities of health professionals and experts [13].

On January 28, 2023, an earthquake with a magnitude of 5.9 on the local wave scale (ML) at a depth of 10 km shook the city of Khoy in the northwest of Iran, killing 3 people and injuring 1167 people. This incident led to the destruction of 9250 residential units, of which 1750 units were destroyed and 7500 residential units were partially damaged [14]. Considering the occurrence of this accident and the special conditions created in the region due to extreme cold and rain, it is important to identify the health needs and challenges of earthquake victims and use these lessons learned to prepare for other possible earthquakes and other disasters. Whereas that no qualitative study has been done regarding the health challenges and people’s needs during the earthquake crisis in Iran, On the other hand we know that provision of services during a crisis is influenced by various individual and organizational factors, along with the prevailing attitudes and culture in society. Therefore, identifying the perceived experiences of people in facing the challenges, along with understanding their responses during the earthquake crisis, plays an essential role in workforce preparation and formulation of strategies to overcome obstacles plays a role, and thus increases the efficiency of emergency services and other agencies in similar situations [15].

Also based on researches, to describe a phenomenon, the most suitable method is a qualitative descriptive approach that focuses on the elements of when, what, where and why it occurs. Qualitative research involves extensive exploration of human experiences and realities, achieved through sustained interaction with people in their natural environments. This approach yields comprehensive and descriptive data that enhance our understanding of these experiences [16]. Therefore, identifying the health challenges that people face during an earthquake is important to address deficiencies and reduce adverse outcomes during a crisis. As a result, this study tries to examine and outline the challenges and health needs of the Iranian society by using the experiences of the Khoy earthquake victims.

## Methods

### Design and setting

This qualitative study employed a conventional content analysis approach in earthquake-affected people in Khoy city located in northwest of Iran. The conventional qualitative content analysis method contains human interpretations and mentalities, and it is the best method to describe perceptions, experiences, and basic social processes [16].

### Participants

This research was conducted using 25 face-to-face interviews with earthquake-affected people in Khoy city. To collect rich information, purposeful sampling was done using the snowball technique to find key informants. The interviews continued until they reached data saturation and achieved a deep understanding of the studied phenomenon. For more diversity of data, the participants were identified and included in the study using the information available in health care centers, medical emergencies, Red Crescent Organization, municipalities, non-governmental organizations, and inquiries from the earthquake-affected urban and rural people of Khoy city.

### Data collection

To collect data, at first, to extract interview themes and develop an interview guide, three unstructured interviews were conducted with people with rich information, and then 25 semi-structured interviews were conducted. The interview guide was used to ensure that all the topics were covered. Interviews were conducted face-to-face with prior arrangements by the interviewers at their workplaces or in one of the camps in the earthquake-affected areas. The interviews continued until data saturation so that no new class or theme was obtained [17]. In this study, data saturation was achieved by up to 20 interviews according to the research group, and to ensure that there are no new data and results, 5 more interviews were conducted.

Before starting the interview, the purpose of conducting the study, how to participate in the interview, and recording the interviews were explained to the participants, and after obtaining informed consent, the interview started. We used interview guide in our study that developed for this study, and has not previously published elsewhere. We uploaded an English language version as a supplementary file. At the beginning of each interview, the demographic information of the participants was asked and then the interview started with a general question. As it was asked to “explain your experience of health challenges after the earthquake”. Probing questions were used according to the interview guide. For example, “What did you need physically and psychologically after the earthquake?“, “What problems did you face after the earthquake?” Also, based on the answers and data analysis, probing interview questions such as “Tell me more about this,” and “What does this mean?” were asked. Duration of each interview was 40 to 60 min. All interviews were recorded using a digital audio recorder (MP3 player). The collection of interviews lasted almost a month and the entire research was conducted between January 2023 and July 2023.

### Data analysis

At the same time as data collection, data analysis was also done with the qualitative content analysis approach using the steps suggested by Graneheim and Lundman [18]. In this way, first, the recorded interviews were written. Then the text of each interview was read several times to get a general understanding of its content. Then the text of each interview was divided into meaning units and each meaning unit was compressed and coded. Then different codes were compared with each other and based on the similarities, differences, and consistency of the content, were placed in sub-categories and main categories. In case of disagreement about the codes, discussion, and exchange of opinions were made by the research team until an agreement was reached about the codes. MAXQDA10 software was used for data analysis.

### Rigor

To achieve the accuracy and trustworthiness of the data, the four criteria suggested by Guba and Lincoln (1985) were considered [19]. For the credibility of the findings, allocating enough time for data collection, and researchers’ long-term involvement with the data was done. For the dependability of the results, the findings were re-reviewed by colleagues and members of the research group. In this way, two colleagues who had qualitative research experience were asked to review the interviews and the initial coding and classes [19]. For the confirmability of the findings, the researchers tried not to interfere with their presuppositions as much as possible in the data collection and analysis, and finally, in terms of the transferability of the findings, it was tried that a detailed description of the process of data collection, stages of coding and their analysis were presented.

## Results

The results related to the demographic characteristics of the participants are presented in Table (1). The findings led to the identification of 104 primary codes, 14 subcategories, and 2 main categories of “management as a missing link in unexpected events” and “emotional actions of people in crisis” (Table 2).

**Table 1:** Demographic characteristics of the participants affected of earthquake

Participants		Hospital staff	Comprehensive urban and rural health centers	Pre-hospital emergency	Red Crescent organization	Municipality	Non-governmental organizations	Earthquake-stricken citizens
ID Number (total 25)		8,15,16,19,23	12,18,22	4,6	3,9,10	1,25	5,11,24	2,7,13,14, 17,20,21
Age	) M ± SD (	39.6 ± 2.7	44.3 ± 1.5	30 ± 2.8	38 ± 1.3	47 ± 2.3	40 ± 1.7	51 ± 1.6
Education	Illiterate	0%	0%	0%	0%	0%	0%	28.5%
	Diploma	0%	0%	0%	0%	50%	0%	14.2%
	Bachelor	60%	100%	66.6%	50%	50%	100%	42.8%
	Masters	40%	0%	33.3%	50%	0%	0%	14.2%
Marital status	Married	80%	66/7%	100%	100%	100%	33.3%	57.1%
	Single	20%	33/3%	0%	0%	0%	66.7%	28.6%
	Divorced	0%	0%	0%	0%	0%	0%	14.3%
Employment status	Housewife	0%	0%	0%	0%	0%	0%	71.4%
	Freelance	0%	0%	0%	0%	0%	66.7%	14.3%
	Employee	100%	100%	100%	100%	100%	33.3%	14.3%
Children number	) M ± SD (	2 ± 0.7	2 ± 1	1.33 ± 1.15	2 ± 0	1.5 ± 0.7	0.33 ± 0.57	2.57 ± 0.7
Work experience	) M ± SD (	13.6 ± 9.8	11.6 ± 3.5	7.33 ± 2.08	16 ± 2.8	5.5 ± 0.7	2.66 ± 0.57	6 ± 2.8
Shelters	Shelter not ventilated and crowded	13.2%	15.2%	9.5%	10%	8.3%	18%	25.8%
	Shelter not warm enough	13.7%	10.7%	9.6%	6.5%	3.5%	19.3%	36.7%
Wash status	Clean water for drinking	98.8%	98.9%	95.6%	85.7%	86.8%	76.5%	69.2%
	Toilets availability	70.3%	93.2%	98.1%	27.8%	58.4%	63.5%	9.2%

**Table 2:** Classes and sub-classes of health challenges obtained from interviews with affected people of earthquake

Category	Sub-category	Some of Codes
Management as a missing link in unexpected events	The challenge of access to emergency resources and health facilities	Poor ventilation in camps.
		Population density in camps
		Lack of tents and safe places for temporary accommodation
		Improper location of shelters
		Need for humanitarian assistance
		Inadequacy of heating devices
		Lack of timely supply of nutritional equipment
		Supplying medicine, equipment and reserves needed for prevention and treatment
		Lack of control of consignments and donated items
		Delayed receipt of facilities
		Temporary assistance
		Lack of bathroom and toilet
	Feeling of abandonment in medical personnel	The need for the support of medical personnel
		The concern of medical personnel for their families
		Non-cooperation regarding medical staff work shifts
		Intercity travel of health workers
	Weakness in the structural safety and infrastructure of the health system	Need to provide a safe place for patients and health workers
		Damage to medical facilities
		The stress of insecure workplace
		Failure to identify safe places in the hospital
		Failure to provide the field hospital on time
		Need to ensure the safety of the hospital’s electrical system
		Weakness in the hospital’s ventilation and heating system
		Low safety of workplace building
	Logistical challenges	Lack of forecasting and preparation for crisis
		Inadequate resources in the warehouse of relief agencies
		Non-availability of patient transfer system
		Lack of ambulances and emergency medical vehicles
	Risk management training	People’s ignorance in the face of crisis
		Ignorance in accommodation and related services
		Inadequate funding for programs to acquire crisis preparedness
		The need for specialized skills of managers and health workers in crisis
	Crisis response management challenges	Lack of urban traffic management
		Failure to establish a central crisis management headquarters
		Failure to manage secondary hazards after an earthquake
		Lack of coordination of organizations and responsible managers
		Improper management in the allocation of health workers
		Need for surplus health workers
		Absence of a quick assessment system for casualties and damages
		The need to send relief teams to the region on time
		The operational commander is unknown
		The description of the duties of relief workers is unclear
		Inappropriate distribution of public aid
		A large crowd of people at the tent distribution site
		Lack of trust in officials in the distribution of relief items
		Self-centered actions in the distribution of public aid
		The need for the presence of health field managers in the process of providing services
		Interruption of the communication system for relief
	Weakness in the intelligent relief system	Disruption in the service computer registration system
		Use of paper form of care report due to slow web-based and computer infrastructure
		Impossibility of timely submission of necessary patient reports
		Incomplete processing of patient information due to manual processing
		Lack of sufficient skills in using the computerized triage system during the crisis
	Management of secondary incidents	Risk of carbon monoxide poisoning
		Risk of fire in temporary accommodation camps and tents
		Risk of food poisoning
		Unsafety of buildings due to structural damage during earthquakes
		The need to protect people’s property and assets
		Insecurity due to the invasion of unknown people in the area
		Low presence of security forces
	Need to provide medical services and disease prevention	Need to visit a doctor inside the camps
		Supplying the medicinal needs of patients
		Supplying the medicinal needs of people with chronic and underlying diseases
		Influenza and cold outbreaks
		Incidence of diarrhea and gastrointestinal diseases
		Lack of doctors to attend the camps
		Need for ambulatory treatment in camps
		Need to take care of pregnant women and children
		Need to treat physical injuries caused by earthquakes
Emotional actions of people in crisis	Overexcitement	Fear of entering the house
		Avoiding the accident site
		The mental concern of repeating earthquakes
		Being stressed in a closed space
		Fear of sleeping alone
		Fear of sound
		Frequent startle
		Heeding excessive alarm about the earthquake
		Fear of an unfortunate incident
		Feeling the emptiness of the world and life
		Occurrence of aggressive behavior
		Need to strengthen tolerance in accidents
		Need to screen people prone to mental illnesses
		Need to send helper and psychological counseling
		Stress caused by loss of home and property
		Need to create a calm psychological atmosphere with people
	Psychological vulnerability of children	Changing children’s eating and sleeping habits
		Excessive attachment to caregivers
		Reduced concentration during games and other activities
		Children’s incompatibility
		Association of earthquake scenes
	Physical complaints caused by stress	Symptoms of heart palpitations, headache, abdominal pain
		Fear of an earthquake while sleeping
		Stuttering and inability to speak
		Numbness on one side of the body
		Need for sedatives
		Imbalance of female hormones
	Confusion caused by the lack of reliable sources of information	Influence of rumors published in cyberspace
		Providing scattered and incorrect information from unreliable sources
		Mistrust and pessimism towards the media
		Ability to accept too much gossip
		Contradiction of the media in providing earthquake-related information
	Negative effects of living together with several families	Loss of privacy inside the tents
		Disruption of family balance
		Social issues for women and girls due to the neighborhood of tents
		Ethical problems inside the camps
		Conflict and violence inside the camps
		Decrease in individual and family independence

### Management of the missing link in unexpected events

#### The challenge of access to emergency resources and health facilities

Stockpiling supplies and first aid should be considered in proportion to the population of the region and its level of vulnerability. The challenge that the participants in the earthquake faced was providing temporary accommodation, relief, and public health needed on a large scale and coordinating their transportation to the affected areas. An example of the participants’ interviews is given below.

“I came to visit this camp, as you see, there is air pollution, noise pollution, excessive and continuous light throughout the day and night, very little distance between tents, windy and humid air, poor air conditioning, and a very high population density. In fact, the number of people is not proportional to the size of the hall and sanitary facilities at all.” (ID number: 12).

“These tents that they gave us are travel tents and not tents that you can sleep outside of your house. This is the Azerbaijan region and now we are right in the middle of winter and the weather is cold until the middle of spring, at least the Red Crescent tents are more suitable. They give us, of course, they give us an electric heater, but the weather is so cold that it does not cover the cold.” (ID number:2).

“All the aid and items that are brought from other cities are not monitored, each region has found a series of people for itself and those people distribute the donations of the people, this has caused our neighborhood to have no materials Food, heating equipment, and clothes do not arrive while another area receives good aid, or whoever is smart quickly goes and takes all the donated equipment for himself and the rest of the people stay.” (ID number:7).

“We have problems with sanitation, electricity, gas and water. The water we bring freezes in the cold outside, we have to light a fire to heat the water, but the smoke from the fire causes the others to protest. For the toilet, we have to walk all the way to get to the gas station toilet or use the toilet inside the hospitals.” (ID number: 13).

“I haven’t bathed in two weeks now, and I go to the office with the same clothes I have on and I didn’t change, because we don’t have bathroom facilities and on the other hand, the weather is cold.” (ID number:14).

#### Feeling of abandonment in medical personnel

Post-disaster planning is divided into three periods of immediate relief, maintenance, and reconstruction, which requires intensive and short-term relief during the period of immediate relief. Most of the medical personnel who participated in the present study admitted that the health officials did not provide any support to their families during this period and their families were left alone, this caused the mental confusion of the medical personnel during the Duties and taking care of patients. Some of the participants’ quotes are shown below.

“My first problem was that unfortunately, the officials did not support me, my family was in the car and I had to provide services in the hospital, none of the officials gave me a tent or a shelter, or told me to leave my family at least in a safe place. May your family rest in peace and then come to the hospital, unfortunately, I didn’t know how to do my own work in the hospital or take care of my family who were alone outside.” (ID number:8).

“My wife and I are both working, we had to take the children to another city because of the earthquake, and we had to go to work from another city every day with our own vehicle and in very bad weather conditions. Let’s come, well, there is always a risk of an accident on the road in the winter season, and on the other hand, we were worried about our children, who were left alone in another city, and no one paid attention to our problems during this time.” (ID number:18).

“I went to a psychiatrist because of the severe stress I was under and he prescribed medicine for me and told me to go on sick leave for a while, I gave it to my manager at the hospital, but they did not accept my physician’s advice for sick leave. They looked at me with mockery, why am I using the medicine, No one understood me or supported me at all.” (ID number:15).

#### Weakness in the structural safety and infrastructure of the health system

The long lifespan of buildings, traditional architecture, the absence of a codified program and the necessary facilities to restore and strengthen the infrastructure of the health system cause safety problems in healthcare buildings. The quotes of some of the participants are listed below.

“Our workplace, which is an operational base, was completely destroyed, and one of our colleagues was also injured and broken. In general, our base was not safe from the beginning, and we had informed the authorities, but unfortunately, they did not pay attention. Now we are in the tent, but is it possible to have an operational base in the tent? At least, they should provide Conex, because the personnel go to different places for operations and no one is here. If the equipment of the base is lost, who is responsible?” (ID number:4).

“I am now at my workplace, but I am worried because this building is old and it is not at all standard. But then I think and say to myself, I have to adapt myself to the situation.” (ID number:16).

“Because the hospital building was very old, we had to completely evacuate the hospital. Most of the families came and took their patients and only those patients who were sick and old and unable to walk stayed, it was a very bad situation because we transferred all the patients in that cold weather and there was no room in other hospitals. We had to keep the patients in the lobby of the hospital. The patients and the staff are all messed up, we don’t even know how many staff and how many patients we have now.” (ID number:19).

#### Logistical challenges

All relief items and equipment need a place for short-term and long-term storage, and since a significant amount of the Red Crescent’s assets are kept in warehouses as relief items and equipment; The management of warehouses requires spending money, so it should be the best performance that guarantees the minimum cost and the welfare of the population when crises occur; to appoint. According to the statements of the participants, one of the challenges they faced during the earthquake crisis was the empty warehouses of the depot, which is mentioned in below, in some of the quotes.

“Well, you see, some issues should have been predicted in advance, everyone knows that Khoy City is located on a fault and we had many earthquakes and aftershocks like this a few months ago. Pre-hospital emergency officials should always have a few sheds ready to use in emergency situations so that if there is a problem with the operational bases, they can quickly transfer the troops to the conexs to do the work, or at least have tents for their own troops beforehand.’ (ID number:6).

“We were not prepared for this crisis; the employees were not trained and the crisis shed was empty of equipment. We don’t have a warehouse in our city so when a crisis or an accident happens, we can go to the warehouse and see what we have to deal with this crisis.” (ID number:4).

“The Red Crescent of our city did not have any facilities, when we called, they were told do not have an ambulance, and they do not even have a blood pressure monitor to check the blood pressure of people who need help, they told us that we have to wait for the equipment to arrive from other cities. Well, the Red Crescent must already have a series of necessary equipment for critical situations.” (ID number:1).

#### Risk management training

Properly informing society about the possibility of a disaster and disseminating expert information to reduce waste and take preventive actions is very important when an earthquake occurs. Some of the quotes from the participants are listed in below.

‘Considering that Khoy City had many aftershocks since the end of summer, should people be taught, what to do when an earthquake occurs? We ourselves had collected information from the Internet, for example, to have packing materials ready, to determine safe places in the house for going there during an earthquake, or to stay next to the pillars of the house, which are strong, and have a phone or a flashlight handy. That if the electricity is cut off, we can use them so that at least we don’t walk over broken glass and places that are unsafe. This was all the information that we told each other and no one taught us.” (ID number:17).

“In my opinion, since this is an earthquake-prone area, the crisis management should be stronger than this. It is possible that an earthquake will occur every year. I expected that a series of practical earthquake drills would be implemented at least for the healthcare workers so that the workers would know what should they do in critical situations? During the earthquake, my colleagues and I were so lost that we didn’t know what to do. We were running away from this side and the patients were running away from the other side, the situation was very confusing.” (ID number:16).

“Well, I have 8 years of service now, but nothing about what to do in a crisis situation. And what is crisis management? They did not tell us anything. Of course, we had an educational crisis program that taught just everything in theory, but we did not conduct a practical maneuver in which the names of the auxiliary forces and the etc. were written, the tasks were clear, and the contact numbers of the people were available. We did not see anything.” (ID number:8).

#### Crisis response management challenges

In many cases, the lack of a written plan to deal with the crisis leads to an incorrect division of tasks between the therapists. Crisis planning helps a lot in keeping the scope of the crisis small and controlling it quickly. The majority of the participants stated that the health team had not taken any action before the crisis, and in fact, they did not have the necessary preparation for the earthquake. So, it caused things to become chaotic. Some of the speakers’ quotes are listed in below.

“In my opinion, our officials themselves did not know what to do, because as the people in charge of the crisis, they must have a mental background of what happened and what they were going to do, but when we asked the officials what to do? Who are you going to call for help? How long is the situation going to be like this?… The assignment was unclear.” (ID number:3).

“Well, the truth is, I think the higher-ups don’t know much about the crisis, we have a flowchart in a crisis, we have an x system, and we have an incident or crisis command system that we have to operate based on, but unfortunately our managers don’t know about any of these. And until we act based on these, we cannot organize the services provided.” (ID number:19).

“I am a medical emergency worker, we had many reports of fights inside the camps, but I did not see anyone from the security and military forces who were present in the camps and took care of these issues.” (ID number:6).

“The crisis committee acted too late, they should have quickly set up camps for the people, they set up tents 7 days after the earthquake, these things should have been done quickly and in the first days. Anyway, they saw people pouring out of their houses, they should have acted quickly on providing tents so that people have a shelter.” (ID number:20).

“My wife and I already had foot aches and now we stayed outside in this cold weather for so long and we slept on the hard and cold ground for so long that now neither of us can walk due to severe foot aches. Finally, after a week we received a tent.” (ID number:21).

“Well, when they call me to come to the hospital, they should also determine my duties in the hospital, what should I do? For example, 10 people should take blood vessels, 10 people should dress the patients, etc. Even though many troops came to the hospital because the duties were not clear, we all went here and there to see where the work was, they must have a clear plan in advance, including who should be called and what their duties are, but really It was not organized.” (ID number:15).

#### Weakness in the intelligent relief system

Pre-hospital emergency information systems provide many benefits if designed and implemented correctly. Research shows that in America, the pre-hospital emergency information system has many capabilities due to more attention to information and communication technology infrastructure, which has led to the integration of the system and better delivery of emergency care. The medical personnel participating in the present study complained about the slow access to patient information in each step of the computerized registration process, from the incident to the transfer of the patient to a medical center. Quotations from contributors in this regard are included below.

“The main factor that slowed down the provision of services to patients during the earthquake was the computer registration of all services and care. It is true that now everything is registered in the system, but it does not work in critical situations, because the electricity was cut off and we had a heavy crowd of people at the emergency department of the hospital. We didn’t know how to send the patient for a scan, how to ask for medicine, etc., so things were going very slowly.” (ID number:8).

“If a separate system is defined for emergency situations, personnel can provide services more easily. Of course, we have the triage system, but if such a system is defined for this situation and colleagues are taught how to use that, it will be very effective.” (ID number:16).

#### Management of secondary incidents

Among the challenges related to health is the occurrence of secondary accidents such as fire, city gas cutoff, gas leakage in houses in the early hours of the earthquake, and the occurrence of carbon monoxide poisoning inside tents and food poisoning. The Quotations of providers are listed below.

“I saw that the weather was very cold, the space inside the tent was small and I couldn’t bring a heating device inside, so I went to get an electric travel heater that was distributed among people. But I didn’t know that when you turn them on for the first time, they have a special smell that causes headaches, after a while I saw that my parents had headaches and loss of consciousness, so I called the emergency and took them to the hospital.” (ID number:13).

“My husband wanted to install the tent, but his foot got stuck on the pole of the tent, and it caused the tent to fall on people and hurt his leg, and we had to admit him to the hospital.” (ID number:7).

“From the camps, called the emergency medical center the donated food, such as pasta and tuna fish, caused them to get food poisoning, or there were too many reports of CO gas poisoning.” (ID number:23).

#### Need to provide medical services and disease prevention

Factors that determine the vulnerability of societies against disasters include the nature and extent of the disaster, population density, and social vulnerability related to poverty, social class, nutritional status and basic health of the affected population, existing health infrastructure before the disaster, the level of access referred to health services and environmental conditions. The participants stated that the crowding of people in the camps had caused the spread of various infectious diseases, and on the other hand, patients with special conditions could not have medical visits as scheduled because doctors’ offices were not open. The quotes of the participants are shown below.

“My child has PKU, which is an enzyme problem. My husband is a taxi driver and he hasn’t been working because of the earthquake, we have almost no money left, my child can’t use normal milk, and most of the pharmacies are closed because of the earthquake, even those were open said that they didn’t have this type of milk and should request the provincial center to come.” (ID number:17).

“Our neighbor has diabetes, and there wasn’t one to take care of him. he had to go to the provincial capital and get his medicines from there, he uses insulin, he went to the pharmacy to get the medicine, but they said that his own doctor should be prescribed the medicine to give her insulin, but nowadays, her doctors is not here, they have to cooperate with him.” (ID number:21).

“The weather here is very cold now, my hands and face are numb from the cold and I got the flu, my children have been sick for a few days now, they say they have the flu, we have a health center in the village, but it has been closed for about 10 days. Any doctors have not come to visit.” (ID number:14).

“The people inside the camps do not need emergency measures, that is, their illness is not such that they need to be admitted to the hospital, and most of them need outpatient measures and prescription of drugs such as antibiotics and painkillers. We have a shortage of doctors and doctors are mobile and may not be present in the camp at that moment. Patients go to pharmacies, but they are also closed or do not have the necessary medicines.” (ID number:23).

### Emotional actions of people in crisis

#### Overexcitement

Timely and quick intervention following the occurrence of an earthquake causes the symptoms caused by a high percentage of survivors to be relieved, and basically, the main task of psychosocial support groups is to provide basic psychological assistance. Some of the participants had problems such as ringing in the ears, insomnia, jump reflexes, and periods of severe stress that forced them to use medicine. Some quotes from the participants are presented below.

“I don’t sleep well anymore, I’m always afraid that there will be happen earthquake again, I wake up early with the slightest sound.” (ID number:13).

“I was very scared. I couldn’t sleep for 72 hours and at the end I was able to sleep a little with the help of sleeping pills. I went to a psychologist, they said that I got PTSD and I have to use medicine for a while, otherwise this disease may continue like this.” (ID number:20).

“I feel insecure and afraid, when I walk, I feel the ground is shaking under my feet, and there is an earthquake again.” (ID number:23).

“There are cracks everywhere in our building, glass windows are broken and dishes are broken and spilled on the floor, that’s why I can’t enter the house at all. I’m scared, I prefer to come out early from the house and not go near it.” (ID number:7).

“If I hear a sound or feel a movement, I am saying that an earthquake has occurred. The earthquake in Turkey had a very bad effect on us, because our city is on the border with Turkey and we felt the aftershocks of the Turkish earthquake here. I keep thinking that if an earthquake of this magnitude comes to our city, or if it lasts a long time like the Turkish earthquake, we would all be trapped under the Debris, and these thoughts increase my stress.” (ID number:14).

“I am a municipal employee, when the earthquake happened, my colleague and I went out of the city to dispose of garbage. I was in the car when there was an earthquake, my colleague had imagined that the mountain that was there during the earthquake completely split apart and stuck together again. He was under so much stress for several days, his tongue was stuck due to the stress and he had urinary incontinence. He felt that the apocalypse had come.” (ID number:25).

#### Psychological vulnerability of children

After an earthquake, the child’s normal living conditions change and the safe environment in which the child’s growth and development take place is destroyed, for this reason, some children experience stress and anxiety. The quotes from the children’s parents are included in below.

“My son’s feeding pattern recently has a problem, he used to eat well, but now he has no appetite, or the form and time of his eating are messed up, for example, he eats breakfast at 12 noon and dinner at 1 am.” (ID number:5).

“I know my son, he hugs me when he is stressed, for example, he used to hug me during school exams, and now I see that he has become like this after the earthquake, I know that because of the fear of the earthquake and his stress is getting closer to me.” (ID number:24).

“That day, the wind came and hit the plastic we put on the tent and it made a hissing sound. I saw that my daughter was scared by the sound of the wind and thought that there was another earthquake. She came to me and said Mother I can’t be alone; I want you to sleep with me. it means that the children are scared to this extent.” (ID number:20).

“My daughter is very scared, she says Mom, I feel that the ground under our feet is like the sea and it is moving like a wave.” (ID number:14).

“Children’s minds are very busy with the earthquake. I saw my daughter telling my son that in our neighborhood, the buildings are ten stories tall and the streets are narrow. If happen an earthquake, how are people going to run out? They will all stay under the debris and die. Why are the buildings so tall and close together?” (ID number:23).

#### Physical complaints caused by stress

Frequent aftershocks and destruction of houses disrupt family order, and due to pressure on families, especially women, they show physical symptoms caused by stress. The physical complaints of the participants are presented below.

“I was so stressed during these aftershocks that my period stopped, I thought I was pregnant, but I went to take a test and they said it was negative. Due to stress, my period cycles are messed up.” (ID number:7).

“I have been taking medicine since a 5-magnitude earthquake occurred about three months ago, and I had just gotten better, but with this 6-magnitude earthquake, my condition worsened again. Therefore, I went to the doctor again and he prescribed Inderal tablets for me and said I have to use them for a while. Now I can’t even stay at home for a minute because I get heart palpitations when I’m alone.” (ID number:2).

“When I entered the house, I panicked, all the walls of the house had collapsed, and the children’s room was completely destroyed, seeing the situation, I felt so much pressure and stress that my left hand started to hurt, I couldn’t even hold the phone in my hand, and everything was messed up. Everyone says that I became like this because of a lot of stress.” (ID number:16).

#### Confusion caused by the lack of reliable information sources

People show sensitivity to rumors according to their personality traits, cognitive characteristics, and vital experiences, and sometimes with curiosity and searching, they find the necessary mental preparation to receive and transmit part or all of the fake news. The participants in the present study expressed their displeasure with the spread of information without an official and reliable source in cyberspace. Some of the participants’ quotes are shown below.

“It’s been 27 days since the earthquake, but we don’t have any reliable news channel from where we can follow the news of the earthquake, there are many rumors spread in cyberspace, but we don’t know if it’s true or a rumor, no one gives us an explanation. Even sometimes the things they say are rumors, we saw them turned into reality and were better than the predictions of the authorities, and earthquakes occurred on the days they had announced on all those dates.” (ID number:13).

“The earthquake in our city was almost at the same time as the earthquake in Turkey. I couldn’t watch the pictures and videos of the Turkish people being pulled out from under the debris. I was so nervous that I cried every time the phone when I opened it, they said that there was going to be an earthquake that would be worse than the Turkish earthquake. Really, all the people of the city were worried and stressed because of all the rumors that were in cyberspace.” (ID number:7).

#### Negative effects of living together with several families

After accidents and natural disasters, problems such as lack of security in camps, non-observance of privacy and religious considerations, veiling, and modesty are common in camps. The participants, especially the women affected by the earthquake, had raised the consequences of not paying attention to their healthy diet, the increase in workload, and daily busyness after the earthquake, such as the need to support children and closely monitor the children of the family. Some also mentioned the possibility of sexual violence remaining hidden in small or rural environments due to the fear of disgrace. The quotes of the participants are listed below.

“Negative effects of multiple households living together. We are gathered together with all the families and live together. It’s true that this has made us a little less stressed, but I can’t reach my children anymore, and I can’t spend much time with my husband. Because we have to observe moral considerations in public. Or we may say something that will hurt others, there are many problems, but one has to bear it, after all, there is a crisis, and we have no choice.” (ID number:17).

“The people who are here together in the camp have no privacy at all because hundreds of tents are put together inside the camp and a hundred strange and different families have been living together for about a month, and this has caused a lack of privacy and sometimes witness moral corruption and sexual problems.” (ID number:11).

“I had to tell the children not to make noise because we were all in a tent with the rest of the family. You could see that there were old people or some of them didn’t like noise and I had to get angry with my children to stop making noise, or I had to tell them from the first day to go outside the tent and play in the cold outside. There is no rest at all and you can’t eat on time.” (ID number:24).

“I feel like I lost my personal life. it is so tight to live in a tent with several other people so you can’t even move your hand while sleeping. I go to work tired now, because I have no rest at all, as if the peace of my life has disappeared, I wonder can I go home again and lie on the couch and watch TV?” (ID number:23).

## Discussion

The current research was conducted using a qualitative method and a content analysis approach to identify people’s health challenges after the earthquake in Khoy city. Considering that the range of threats to public health around the world is wide and diverse and includes the spread of infectious diseases, food and water, chemicals and radiation pollution, natural and technological hazards, and climate change, help to face these threats and other challenges related to them, countries should try to strengthen their capacity to manage disasters, especially natural disasters, through prevention, mitigation, preparedness, response and recovery measures. Effective preparation, response, and return of society to the normal situation before disasters require detailed planning and integrated and coordinated efforts of professional and experienced staff in applying knowledge and management skills in critical situations [20].

### Management as a missing link in unexpected events

One of the important subcategories that existed in the results was the challenge of accessing emergency resources and health facilities. In the big earthquakes of the last two decades in Iran, a large number of victims were completely confused due to the lack of evacuation centers and emergency accommodations and even spent nights without shelter in the cold [21]. The study by Najafi et al. (2020) in Iran also showed that many participants thought that emergency accommodation and aid have challenges such as inconsistency in the distribution of tents and facilities, the preference of people and even government institutions for self-centered measures in the distribution of goods and creation of heavy traffic, the loss of many donated items due to improper distribution or storage, lack of attention to the dignity of the survivors in the distribution of items, and the lack of transparency in the collection and distribution of public aid were associated with it [22]. According to the results, one of the main elements in crisis management planning is the management of the settlement process and the distribution of public aid before the occurrence of natural disasters.

Another subclass was the weakness of the infrastructure of the health system. In the study of Suganuma et al. (2006), creating earthquake-resistant buildings preventing the construction of structures without using earthquake criteria and standards, and revising building standardization rules, are discussed in the discussion of resilience. It should be mentioned that the retrofitting of structures does not include only residential buildings and all structures should be taken into consideration [23]. Some worn-out tissues in the health system and hospitals need special attention in the city area and need detailed planning in the crisis management system to improve the safety of patients and employees. The efficiency and dynamics of the crisis management system and the provision of safety and relief services to citizens living in these contexts require special conditions [24].

The feeling of abandonment of medical personnel was among the other subcategories obtained in the present study. Medical personnel expressed dissatisfaction with the indecision and abandonment of their families during the intensive and short-term process of providing relief and services to the earthquake-affected people. They expected the treatment network to at least support their families during this time. Seligman (1975) states that if people have significant experiences that show that whatever they do does not affect what happens to them, the expectation is formed that their actions will generally have no beneficial results for them. It will not work, so they will lose their ability to perform beneficial behavior in other situations. These people may lose their motivation and become anxious and depressed [25].

Stockpiling supplies and first aid should be considered in proportion to the population of the region and its level of vulnerability. In case of insufficient stockpiling, it is possible that the lives of some people, such as children, may be taken due to food restrictions, or the injured may die due to medical restrictions. This challenge was one of the subcategories obtained in the present research. According to the comprehensive guidelines for the relief warehouses of the Iranian Red Crescent Society, all relief items and relief equipment need a building and a place for short-term and long-term storage, and since a significant amount of the assets of the Red Crescent Society as relief items and relief equipment and Salvage is kept in warehouses; Management of warehouses requires spending money, so it should be the best performance that includes the minimum cost and the welfare of the population; to appoint [26].

To reach an acceptable level of earthquake preparedness, extensive education through audio-visual devices, published books, and posters, regarding the reality of the earthquake and its consequences is necessary [27]. Therefore, one of the obtained subcategories is risk management training.

Özer (2023), acknowledged that clarifying perceptions and increasing local knowledge through education is one of the effective factors in people’s responses to natural hazards [28]. Carter et al. (2016) in research on the perspective of resilience indicators in the United States mention education and knowledge as one of the tools and international policies to increase the resilience of communities and reduce the risk of accidents [29].

As an earthquake-prone country, China has trained tens of thousands of earthquake experts and has trained and launched a coherent system under the title of crisis management and rapid response. In Japan, during the past 120 years, a huge collection of seismological knowledge, law and regulation compilation, crisis management and compensation institutions, and urban planning patterns have been provided. In these countries, the training of urban managers to face the crisis and supervise construction is considered one of the most serious and urgent measures [30].

Crisis preparation and recognition is one of the duties of management, but more important than that is predicting the crisis and taking the necessary measures when it occurs. Since in the present study, the health management team was not prepared to deal with this crisis, the helplessness of the rescue management team was another subclass. Avizo et al. (2018) proposed that having an operational plan for all types of crises that occur in the field of activity, along with tactical and strategic guidance groups, can change the situation completely. This provides the best possible opportunity to deal quickly and decisively with any crisis that occurs. An emergency plan that is specially prepared for the organization and its specific problems can prevent the growth of the crisis [31]. Considering the occurrence of earthquakes in the four countries of Japan, India, Iran, and Turkey and the history of dealing with this phenomenon, and considering the high statistics of earthquake casualties in Iran as the most important natural crisis, it seems that it is possible to learn from the experience of these countries used to develop crisis management in Iran.

The lack of a separate intelligent assistance system was another subclass of the present study. Pre-hospital emergency information systems provide many benefits if designed and implemented correctly. Research shows that in America, the pre-hospital emergency information system has many capabilities due to more attention to information and communication technology infrastructure, which has led to the integration of the system and better delivery of emergency care [32]. However, most of the current systems are often not responded due to relying on manual mechanisms or lack of design according to the needs, and have many limitations and challenges, including recording the data of emergency patients in isolation and not sharing information at the appropriate time.

Chan et al. (2011) in using an electronic system to record data, concluded that the recorded clinical data such as identity information of the patients, the severity of the disease, the information related to the triage department, the transfer status of the patients and their tracking were significantly better than the control group that used paper forms for documentation [33].

In his study, Greene emphasized that timely information is critical in an emergency, he stated that if hospital providers in an emergency cannot access the patient information within an hour of arriving from the pre-hospital emergency, that information has no value in treating the patient. They mentioned the solution to this problem is the use of electronic systems and the simultaneous presentation of information [34].

One of the subcategories obtained in the present study was secondary incident management. The studies shown earthquake survivors that the major incidents that occur during an earthquake crisis are fires caused by damage to the fuel transmission network and collisions with high voltage power lines [35]. They are hospitalized due to food poisoning, limitation of food sources and disruption of food preparation and distribution methods, lack of sanitary detergents, power outages, and lack of access to refrigerators to store perishable food are among the factors that put food safety at risk in the earthquake-affected areas [36].

During earthquake relief, food inspection is done only based on the appearance, physical conditions, taste and smell of the food, etc., and in these conditions, it will be difficult to control the food effectively. Therefore, previous planning to respond to emergencies during an earthquake requires the compilation of management plans such as policies, requirements, and instructions related to food supply in response to unforeseen conditions and recovery and relief operations That this important thing needs to be anticipated and prepared by the health and treatment organizations at the community level and other service organizations (Red Crescent), If any negligence or weakness occurs in this matter, it will certainly cause an escalation in the crisis [37].

The need for health and medical services was one of the other challenges of earthquake-affected people in the present study. Normally, when disasters occur, due to violence and insecurity, population displacement, and the collapse of the healthcare system, the health status of the society becomes very inappropriate. Damaged infrastructure caused by disasters may disrupt the provision of normal health services, vaccination, and care for chronic diseases in the community for months or even years [38]. The study of Safarpour et al. (2020) also showed that the transmission of diarrheal diseases, dehydration, acute respiratory infections, meningitis, measles, tetanus, malaria, etc. increases due to poverty and population density, especially in refugee camps [39]. Therefore, when disasters occur, the healthcare system becomes a very prominent and important element, which is necessary for the immediate health response and the recovery and reconstruction phase of crisis management [40].

### Emotional actions of people in crisis

One of the main categories obtained from the results of the present study was the emotional actions of people in crisis. Disasters can cause a lot of psychological stress. Health and mental health problems are a major public health concern following disasters. Lack of mental health services or increased stress may lead to increased suicide attempts, domestic violence, and feelings of anxiety [38]. Therefore, screening by mental specialists is necessary to look for specific symptoms among family members and to provide them with psychosocial support. During the screening, one should ask about symptoms such as sleep disturbance, difficulties in remembering the incident, intrusive thoughts that the person cannot get rid of, symptoms of hyperarousal and irritability, and cognitive and behavioral avoidance in a way that people can understand. If any of these symptoms are present in the person, the person can be included in the support group [41].

Based on the results, overexcitement was one of the important subcategories obtained in the present study. Individuals may show increased psychological arousal after traumatic events. Therefore, they may be nervous, restless, and anxious, and panic very quickly. They may become hypersensitive, experience tinnitus, overdose on meperidine, or have problems concentrating. The study by Wakashima et al. (2019) showed that a person’s performance in terms of friendships and family relationships, and occupational and academic performance is strongly affected by hyperarousal [42]. There are sufficient evidence indicates the increase of this type of psychological arousal about experiencing disturbing memories [43]. In the researcher’s opinion, teaching relaxation skills plays an essential role in preparing a person to gradually face the factors that cause hyperarousal.

All the symptoms and disorders that exist in adults also occur in children. The main difference between children and adults is that children are more vulnerable due to the loss of adult support and the loss of primary caregivers. Therefore, one of the subcategories obtained in the present study was children’s psychological vulnerability. Roysircar et al. (2021) highlighted that children show different symptoms based on their age and developmental stage. Vulnerability in children is in the form of crying, various sleep disorders, bedwetting, problems in feeding, aggression, problems in concentration, as well as learning disorders, academic problems, and fear of repeating the unfortunate event and regressing to previous developmental stages [44]. Therefore, paying special attention to the needs of children immediately after the accident and trying to solve them in the shortest time and in the right way is effective in reducing the level of vulnerability and psychological conflicts in the future.

Usually, the number of physical complaints is higher in communities that survived an unexpected accident. In the present study, physical complaints caused by stress were obtained as one of the subcategories of the research. According to the results of previous studies, under normal conditions, 20–30% of adult patients referring to health and treatment centers have pseudo-physical problems. This rate increases significantly after unexpected events and psychological symptoms and reactions may appear physically. One thing that should be noted is avoiding unnecessary diagnostic measures in these patients because such an approach will stabilize the disease in them [45, 46]. Due to the referral of these people to non-psychiatric doctors and specialists, the training of this category of healthcare workers is of particular importance.

One of the things that were in the interviews of most of the participants was “confusion caused by the lack of reliable sources of information”. The rumors related to the earthquake were spread in cyberspace and caused negative effects such as increasing anxiety and worry among people. The study of Solhi et al. (2022) emphasizes that news and rumors about the earthquake and images of injured or dead people are broadcast for weeks on television channels and social networks without censorship [47]. Although the main cause of trauma is experiencing the moment of the earthquake, continuous exposure to images and the spread of different opinions about the occurrence of aftershocks also prolong the duration of trauma [47, 48]. Of course, according to the researchers of the current study, by using false media such as rumors, it is possible to apply correct management and control the scope of the rumor, in addition to highlighting and reminding the reality of the city’s seismicity, the positive impact of the rumor in the direction of increasing the safety culture in It was used against the earthquake and the level of preparedness of relief organizations.

The negative effects of multiple households living together were among the other subcategories obtained in the present study. In temporary accommodation centers, security, privacy, and family needs are neglected [31]. The results of the qualitative study by Yoosefi et al. (2021) showed that the disruption of order, creating insecurity, the breakdown of social relations, and on the other hand, women being pressured to provide food and shelter in emergencies can expose them to sexual abuse. Put that the result is the occurrence of mental disorders, an increase in cases of unwanted pregnancies, and sexually transmitted diseases [49]. Of course, in the present study, many sexual problems and some cases of sexual misconduct and extramarital relationships in the region were not reported due to cultural factors and remained silent.

### Limitations

One of the limitations of this study was the challenge in planning and coordinating interviews with the participants. This was due to the confusion of the participants in the early days of the earthquake, and the participants needed mental stability to conduct the interviews, which created problems in planning and ensuring that all interested people participated in the study. Another limitation is related to the limited nature of the data, which relies on information provided by participants selected through purposive sampling, with an emphasis on those with the most experience. As a result, the perspectives of less experienced individuals, who potentially present distinct challenges, may not be fully explored. In addition, the data exclusively reflect the experiences of the participants involved in a specific geographic and cultural region, so the needs and challenges may be different in other regions.

## Conclusion

According to the results of this study the range of public health challenges in the earthquake crisis is wide and very diverse, to help deal with these threats and other challenges related to them, countries should try to improve their capacity to manage disasters, especially natural disasters. To summarize, in response to an earthquake, it is necessary to prioritize the following measures: Provide adequate, safe and warm shelter for vulnerable groups. distribution of non-food items and provision of food; Addressing health service gaps, including providing medical care, psychosocial support, ensuring coordination of assistance and resources; Addressing tensions and protection issues in shelters; addressing educational needs; addressing accessibility issues and the need for assistive devices; and addressing the long-term needs of affected communities. Efforts to maintain access to preventive health services in disasters and basic preparedness measures including identification of suitable managers, planning and training of health workers, and preparation of health care systems at different levels of society are necessary to reduce adverse health effects in disasters. In this way crisis managers should training about prevention programs such as immediate response, reduce damage and recovery. Psychological preparation, effective response of people and return of society to the normal situation in disasters require detailed planning and integrated and coordinated efforts of specialized and experienced professional staff in applying skills in critical situations.

## Supplementary Information

Supplementary Material 1.

## Data Availability

Data are available upon request to the corresponding author.

## Abbreviations

EMS: Emergency Medical Service
CPR: Cardiopulmonary Resuscitation
