# Supplementary material for: Exploring the health challenges of affected people in the 2023 Khoy earthquake: a content analysis
Source: BMC Emerg Med. 2024 Oct 28;24:204. doi: 10.1186/s12873-024-01114-7 (PMC11514777; doi:10.1186/s12873-024-01114-7)
Supplement: Supplementary file 1 — Supplementary Material 1. [file 12873_2024_1114_MOESM1_ESM.doc]

**Semi-structured interview guide**

1. **Basic interview questions**

When you hear the word "earthquake", what comes to your mind?

Describe how you feel about the earthquake.

What did you do when earthquake happened?

Explain your experience since the beginning of successive earthquakes in Khoy city.

Explain your experience of health challenges after the earthquake.

What did you need physically and psychologically after the earthquake?

What problems did you face after the earthquake?

1. **Probing interview questions**

Can you explain more about this?

Do you continue this discussion by mentioning a good memory and a bad memory?

What do you mean when you say...?

Do you know another example in this field?

Would you like to discuss another topic?

Do you mean...?

Is it true that you feel that...?

Do you think there is a connection between... and...?
